# IL-1α and Complement Cooperate in Triggering Local Neutrophilic Inflammation in Response to Adenovirus and Eliminating Virus-Containing Cells

**DOI:** 10.1371/journal.ppat.1004035

**Published:** 2014-03-20

**Authors:** Nelson C. Di Paolo, Lisa K. Baldwin, Eric E. Irons, Thalia Papayannopoulou, Stephen Tomlinson, Dmitry M. Shayakhmetov

**Affiliations:** 1 Lowance Center for Human Immunology, Departments of Pediatrics and Medicine, Emory University School of Medicine, Emory University, Atlanta, Georgia, United States of America; 2 Division of Medical Genetics Department of Medicine, University of Washington, Seattle, Washington, United States of America; 3 Division of Hematology, Department of Medicine, University of Washington, Seattle, Washington, United States of America; 4 Department of Microbiology and Immunology, Medical University of South Carolina, Charleston, South Carolina, United States of America; 5 Ralph H. Johnson Veterans Affairs Medical Center, Charleston, South Carolina, United States of America; University of Michigan, United States of America

## Abstract

Inflammation is a highly coordinated host response to infection, injury, or cell stress. In most instances, the inflammatory response is pro-survival and is aimed at restoring physiological tissue homeostasis and eliminating invading pathogens, although exuberant inflammation can lead to tissue damage and death. Intravascular injection of adenovirus (Ad) results in virus accumulation in resident tissue macrophages that trigger activation of CXCL1 and CXCL2 chemokines via the IL-1α-IL-1RI signaling pathway. However, the mechanistic role and functional significance of this pathway in orchestrating cellular inflammatory responses to the virus *in vivo* remain unclear. Resident metallophilic macrophages expressing macrophage receptor with collagenous structure (MARCO^+^) in the splenic marginal zone (MZ) play the principal role in trapping Ad from the blood. Here we show that intravascular Ad administration leads to the rapid recruitment of Ly-6G^+^7/4^+^ polymorphonuclear leukocytes (PMNs) in the splenic MZ, the anatomical compartment that remains free of PMNs when these cells are purged from the bone marrow via a non-inflammatory stimulus. Furthermore, PMN recruitment in the splenic MZ resulted in elimination of virus-containing cells. IL-1α-IL-1RI signaling is only partially responsible for PMN recruitment in the MZ and requires CXCR2, but not CXCR1 signaling. We further found reduced recruitment of PMNs in the splenic MZ in complement C3-deficient mice, and that pre-treatment of IL-1α-deficient, but not wild-type mice, with complement inhibitor CR2-Crry (inhibits all complement pathways at C3 activation) or CR2-fH (inhibits only the alternative complement activation pathway) prior to Ad infection, abrogates PMN recruitment to the MZ and prevents elimination of MARCO^+^ macrophages from the spleen. Collectively, our study reveals a non-redundant role of the molecular factors of innate immunity – the chemokine-activating IL-1α-IL-1RI-CXCR2 axis and complement – in orchestrating local inflammation and functional cooperation of PMNs and resident macrophages in the splenic MZ, which collectively contribute to limiting disseminated pathogen spread via elimination of virus-containing cells.

## Author Summary

Adenovirus (Ad) induces a potent activation of pro-inflammatory cytokines and chemokines upon interaction with tissue macrophages *in vivo*. However, critical factors affecting cellular inflammatory responses to Ad and their functional significance remain unclear. Here we show that in the model of disseminated infection, intravenous Ad administration leads to a rapid release of pro-inflammatory Ly-6G^+^7/4^+^ leukocytes (PMNs) from the bone marrow into the blood. PMNs enter into peripheral tissues and, in the case of spleen, are accumulated in proximity to the virus-containing MARCO^+^ macrophages within the splenic marginal zone (MZ). Mechanistic dissection of molecular queues that guide PMN migration reveals that CXCL1 and CXCL2 chemokines are only partially responsible for CXCR2-dependent PMN recruitment into the splenic MZ. We further found that complement cooperates with IL-1α-IL-1RI-CXCR2 signaling pathways in recruitment of PMNs to the splenic MZ, which results in elimination of virus-containing MARCO^+^ macrophages from the spleen. Administration of complement-blocking CR2-Crry or CR2-fH proteins into IL-1α-deficient, but not wild-type, mice prevents PMN accumulation in the splenic MZ and elimination of virus-containing macrophages from the spleen. Our study defines the functional significance of molecular and cellular host defense mechanisms that cooperate in eliminating virus-containing cells in the model of acute disseminated Ad infection.

## Introduction

Viral vectors based on adenovirus of human and animal types have been widely adapted for gene transfer applications both *in vitro* and *in vivo*. Adenovirus (Ad) is remarkably efficient at infecting both dividing and non-dividing cells and its non-enveloped capsid tolerates major modifications that may restrict or target virus entry into desired cell or tissue types [1]. In permissive cell types, Ad replication follows the lytic cycle, which culminates in productive virus genome replication and release of progeny virions upon cell lysis. This feature of the virus is adopted in the concept of oncolytic Ad vectors, where through specific genetic modifications of the viral genome, virus replication and cell lysis are restricted to cancer cells [2]. Although in preclinical and clinical studies local delivery of oncolytic Ad was found to be safe, intravascular administration of Ad vectors, especially at high doses, activates host innate immune and inflammatory responses that may result in morbidity and mortality [3], [4], [5], [6]. Molecular mechanisms responsible for the activation of severe innate immune and inflammatory responses to Ad remain poorly understood.

Macrophages are the first line of cellular defense against invading pathogens. Tissue resident macrophages in the liver (Kupffer cells), spleen (macrophages expressing macrophage receptor with collagenous structure, MARCO^+^, in the splenic marginal zone), and lung sequester Ad particles after their intravascular administration [7], [8], [9], [10]. These cells are believed to be the principal activators of inflammation in response to Ad since in macrophage-depleted mice, Ad administration leads to greatly reduced inflammatory responses, compared to control un-manipulated animals [7], [11]. Using gene deficient mice, we previously showed that inflammatory cytokines and chemokines were activated by Kupffer cells in the liver and MARCO^+^ macrophages in the spleen as early as 10 minutes after intravenous Ad administration [10]. We further showed that IL-1α-IL-1RI was a key pathway driving inflammatory cytokine and chemokine activation at this early time point after virus injection [10]. Despite our better understanding of the molecular mediators of inflammation triggered by Ad *in vivo*, their contribution to orchestrating cellular host inflammatory responses to the virus remains unclear.

In this study, we analyzed the functional consequences of activation of IL-1α-IL-1RI-dependent and independent pathways in triggering cellular inflammatory responses to Ad in a model of acute disseminated infection in mice. We found that the IL-1α-IL-1RI-CXCR2 signaling axis cooperates with complement to recruit Ly-6G^+^7/4^+^ polymorphonuclear leukocytes (PMNs) to the splenic marginal zone in the proximity of virus-containing MARCO^+^ residential MZ macrophages. This PMN accumulation in the splenic MZ is associated with elimination of MARCO^+^ macrophages from the spleen. Pharmacological inhibition of complement activation in IL-1α-deficient mice prevents PMN recruitment to the splenic MZ and elimination of virus-containing macrophages. Our study reveals non-redundant roles of distinct molecular components of the innate immune response - IL-1α-IL-1RI-driven activation of CXCL1 and CXCL2 chemokines and complement - in enabling recruitment and functional cooperation of cellular components of innate immunity for elimination of virus-containing cells in the model of acute disseminated Ad infection.

## Results

### Intravascular Ad administration triggers the egress of polymorphonuclear leukocytes from the bone marrow

**Figure 1:**
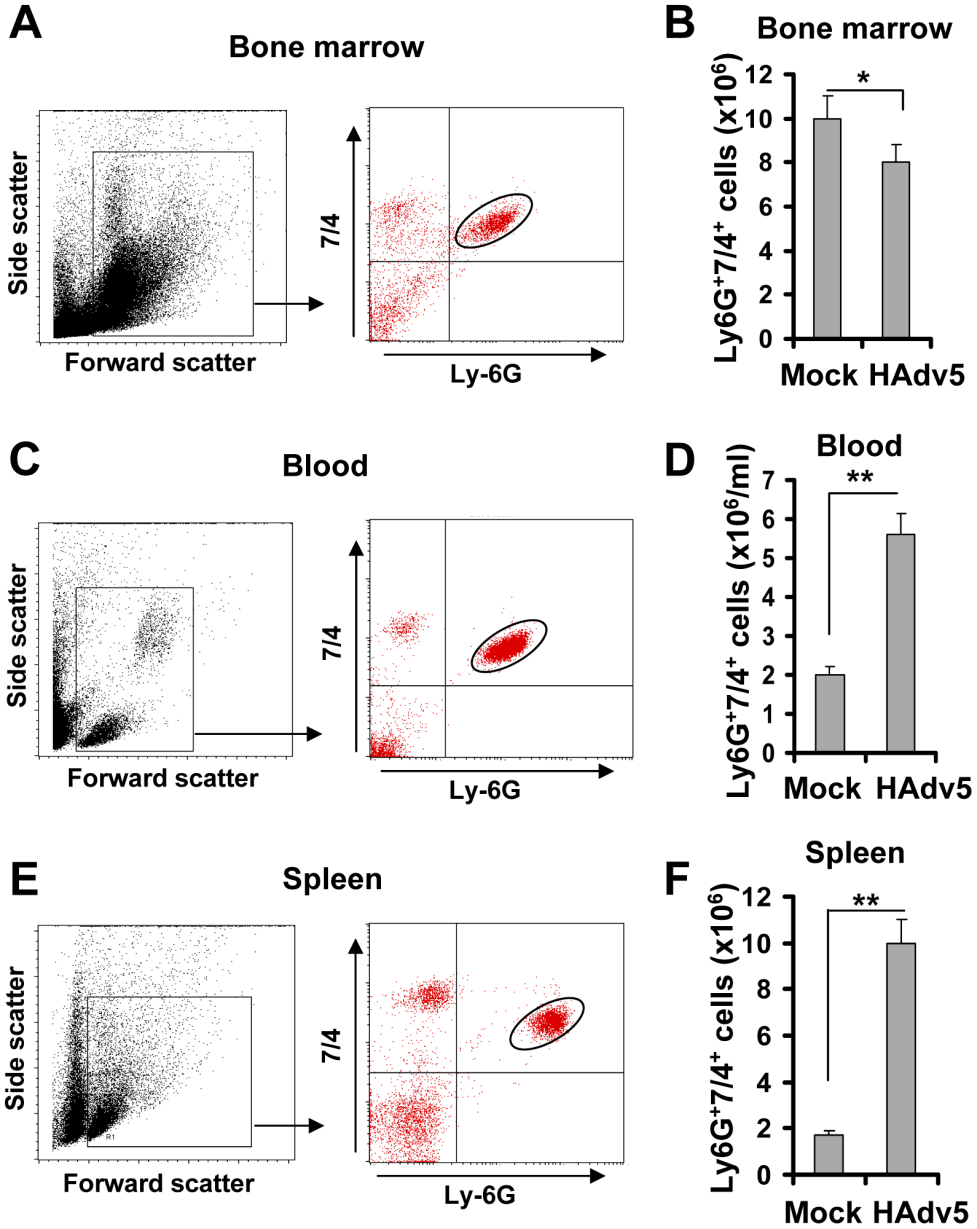
Intravascular adenovirus administration leads to the release of Ly-6G^+^7/4^+^ polymorphonuclear leukocytes from bone marrow into the blood and their retention in the spleen. Dot-plot of the forward and side light scatters of leukocytes in the bone marrow (A), blood (C), and spleen (E) and their analysis for expression of Ly-6G and 7/4 cell surface marker using flow cytometry. The representative dot-plots, obtained from five mice are shown. The population of cells that stained positive for both markers is depicted on the right panel with an oval. Quantification of Ly-6G^+^7/4^+^ cells in the bone marrow (B) (the absolute numbers shown are for one femur bone), blood (D) and spleen (F) of mice injected with saline (Mock group) or Ad vector (HAdv5 group). The data was collected from three independent experiments, N = 5. * - P<0.05; ** - P<0.01.

Intravascular injection is a preferred delivery route for viral gene transfer vectors that are aimed at targeting specific disease-affected tissues or disseminated cancer cells *in vivo*. In clinical settings, disseminated Ad infections with virus titers reaching as high as 10^8^ to 10^10^ virus particles per ml of blood can also be observed in a subset of immunocompromized patients undergoing bone marrow transplantation [12], [13], [14], [15], [16]. To analyze the acute cellular host responses to intravascular virus injection, we infected wild type mice with slightly lower amounts of HAd5-based vectors (5×10^9^/ml of blood, which is equal to 10^10^ virus particles per mouse). This and higher virus doses are known to induce strong inflammatory cytokine production [3], [17], [18]. Two hours after virus injection, mice were sacrificed and the cellular compositions of bone marrow, blood, and spleen were analyzed by flow cytometry. Neutophils are the polymorphonuclear leukocytes of the myeloid lineage (PMNs), and they are among the most abundant leukocytes in the peripheral blood and the bone marrow that promptly respond to tissue injury or infection by migrating to potential infection sites and deploying an array of effector factors that aim at limiting invading pathogens [19]. The analysis of the number of inflammatory PMNs (that can be identified by staining with antibodies recognizing Ly-6G and 7/4 cell surface markers [20]) in the bone marrow after saline (Mock) or Ad injection showed that there was a slight, but significant reduction in the absolute number of PMNs in the bone marrow after virus administration, compared to the control mock-injected group (Fig. 1A–B). Considering that the number of total bone marrow cells in one femur bone of a mouse is equivalent to 6.7% of total marrow cells in the mouse body, the detected egress of 2×10^6^ Ly-6G^+^7/4^+^ cells from the bone marrow harvested from one femur bone is equivalent to 30×10^6^ PMN cells leaving the marrow and entering the bloodstream and peripheral sites in response to Ad administration. In agreement with this assumption, the analysis of the blood and spleen for the presence of Ly-6G^+^7/4^+^ PMNs showed that the absolute numbers of these cells were greatly increased in both peripheral blood and spleen after Ad injection (Fig. 1). Although it was previously demonstrated that PMNs enter peripheral tissues (such as liver) after intravascular Ad administration [17], [21], [22], the quantitative analysis of leukocyte populations of the liver is technically challenging. In contrast, the methods to analyze cellular composition of the spleen are well established, therefore reliable quantitative data can be obtained by using both immuno-fluorescence and flow cytometry approaches. Using flow cytometry analysis, we found that the number of Ly-6G^+^7/4^+^ cells in the spleen increased 5-fold, compared to saline-injected animals, and reached the absolute number of 10×10^6^ cells after injection of mice with Ad (Fig. 1E–F). This data indicates that at the time of analysis (2 hours after the virus injection), one-third of PMNs that have been released from the bone marrow, were detectable in the peripheral blood, one-third in the spleen, and the remaining cells are likely to be distributed among other peripheral tissues.

**Figure 2:**
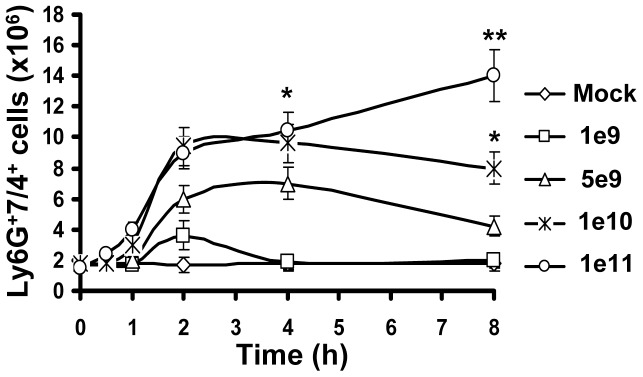
Kinetics of Ly-6G^+^7/4^+^ leukocyte recruitment and retention in the spleen in response to intravascular administration of adenovirus. Accumulation of Ly-6G^+^7/4^+^ cells in the spleen over time after injection of mice with escalating doses of Ad, shown as virus particles per mouse. Data obtained from three independent experiments are shown. Mock mice were injected with phosphate-buffered saline (PBS) only. Splenocytes were harvested, stained with antibodies for Ly-6G and 7/4 cellular markers and analyzed by flow cytometry. N = 4. * - P<0.05; ** - P<0.01. Error bars represent standard deviation of a mean.

To analyze whether accumulation of PMNs in the spleen was virus-dose- and/or time-dependent, we injected mice with Ad at doses ranging from 10^9^ to 10^11^ virus particles (vp) per mouse and harvested spleens for flow cytometry analyses from 0 hours to 8 hours after virus administration. This analysis showed that when Ad was injected into mice at doses ranging from 10^9^ to 10^10^ vp per mouse, the number of PMNs in the spleen increased rapidly, reaching its maximum at 2 hours post virus injection, and then steadily declined by 8 hours after the virus administration (Fig. 2). At a virus dose of 10^11^ vp per mouse, the number of PMNs continued to increase at all time points analyzed and at 8 hours after virus injection the proportion of PMNs in the spleen of mice injected with this dose of virus was four-fold greater than mice administered with 10^9^ vp and two-fold greater than in mice administered with 10^10^ vp/mouse.

Taken together, these analyses revealed that in response to intravascular Ad administration, PMNs with a Ly-6G^+^7/4^+^ pro-inflammatory phenotype are rapidly released from the bone marrow into the blood. The accumulation of PMNs in the spleen was virus dose dependent, with the highest number of cells observed at 2 hours after the virus injection for the doses of up to 10^10^ vp/mouse. At this time point, one-third of the total Ly-6G^+^7/4^+^ cells released from the marrow was recovered from the spleen.

### Recruitment of PMNs in the splenic MZ occurs in response to local pro-inflammatory stimuli

**Figure 3:**
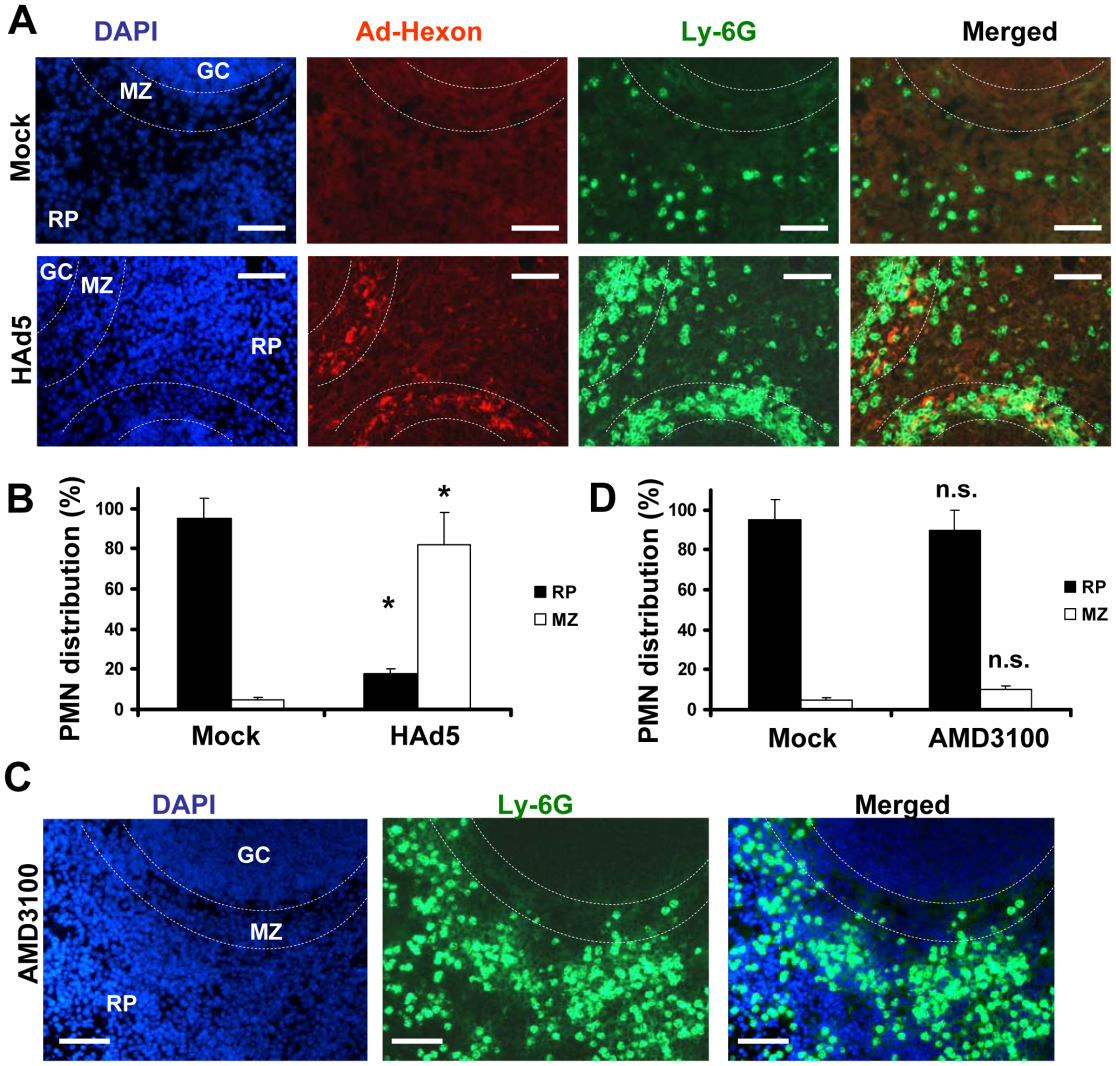
Specific Ly-6G^+^ leukocyte retention in the splenic marginal zone occurs in response to Ad, but not AMD3100. (A) Distribution of Ly-6G^+^ cells (green, FITC-labeled anti-Ly-6G antibody) on sections of spleen harvested from mice that were injected with either Ad or saline (Mock panels) 2 hours after treatment. The splenic marginal zone (MZ), located between the germinal centers (GC) and red pulp (RP) anatomical compartments, is outlined with dotted lines and was defined based on section staining with DAPI (blue). MZ macrophages trap Ad particles from the blood (Ad-Hexon antibody staining, red). Representative panels are shown. N = 5. Sections at three depth levels were obtained from each mouse. (B) Quantitative representation of PMN distribution on sections of the spleen 2 hours after virus challenge. Ly-6G^+^ cell (PMN) distribution was analyzed on at least 4 consecutive sections of the spleen, cut at three depth levels from each individual mouse. The pictures of spleen sections with average PMN cell densities were taken and up to 200 total PMN cells were counted and assigned to MZ or RP compartments based on their actual localization. For each experimental condition, the data were combined and averaged from three to 5 individual mice. * - P<0.05. Mock – mice injected with saline. (C) Distribution of Ly-6G^+^ cells (green, FITC-labeled anti-Ly-6G antibody) on sections of spleen harvested from mice that were injected with small molecular drug AMD3100 2 hours after treatment. The splenic marginal zone (MZ), located between the germinal centers (GC) and red pulp (RP) anatomical compartments, is outlined with dotted lines and was defined based on section staining with DAPI (blue). Representative panels are shown. N = 5. Sections at three depth levels were obtained from each mouse. (D) Quantitative representation of PMN distribution on sections of the spleen 2 hours after the mouse was challenged with small drug AMD3100. The cellular localization was assessed and analyzed as described in (B). n.s. – not statistically significant, compared to corresponding control group. RP – red pulp; MZ – marginal zone.

The spleen is the largest secondary immune organ in the body and is responsible for initiating immune responses to blood-borne antigens and for filtering the blood of old and damaged red blood cells. The spleen is comprised of two functionally and anatomically distinct compartments, the red pulp and the white pulp. The red pulp is a blood filter that removes foreign material and damaged erythrocytes. The white pulp is composed of three subcompartments: the periarteriolar lymphoid sheath (PALS), the follicles, and the marginal zone (MZ) [23], [24], [25]. Blood flowing through the marginal sinus and marginal zone percolates through the marginal zone in the direction of the red pulp. Previously, we had shown that after intravascular administration, Ad particles are sequestered in the spleen specifically by the MARCO^+^ marginal zone macrophages [10]. To analyze whether Ly-6G^+^7/4^+^ PMNs localize to a particular anatomical compartment in the spleen after Ad injection, we injected mice with Ad and analyzed distribution of PMNs on spleen sections 2 hours after virus injection. In agreement with previous findings [10], staining of spleen sections with anti-Ad hexon antibody demonstrated that the vast majority of the hexon-specific staining localized to the splenic marginal zone (Fig. 3A). Staining of the spleen sections with Ly-6G-, Gr-1-, or 7/4 PMN marker-specific antibodies revealed identical patterns of distribution of positive cells, and the vast majority of these cells were also localized to the marginal zone, and not to other splenic anatomical compartments (Fig. 3A and B). It is worth noting that when mice were injected with saline, less than 5% of PMNs were localized to the splenic MZ (Fig. 3A, Mock group), suggesting that PMN recruitment in the MZ may constitute a spleen-specific host response to Ad administration.

To further delineate whether PMN localization to the MZ after Ad administration is specific and not merely a reflection of the increased number of circulating Ly-6G^+^7/4^+^ cells in the peripheral blood, we purged monocytes from the bone morrow via a non-inflammatory stimulus by using the small drug AMD3100 that interferes with SDF1-CXCR4-mediated retention of PMNs and stem/progenitor cells in the bone marrow [26], [27], [28], [29]. In agreement with previous findings, administration of AMD3100 resulted in a massive release of mature leukocytes, including Ly-6G^+^ cells, from the bone marrow into the circulation and peripheral tissues (Fig. 3C). However, the accumulation of these cells in the splenic MZ was not different from that observed in saline-injected mice, with less than 8% localizing in MZ (Fig. 3D). These data provide direct evidence that under local non-inflammatory conditions, the traffic of Ly-6G^+^ cells through the spleen does not result in their accumulation in the MZ. These data further suggest that entry and retention of PMNs in the splenic MZ in response to Ad is specific and is not merely a reflection of passive accumulation in the spleen from a circulating granulocyte pool in the blood observed after virus administration.

**Figure 4:**
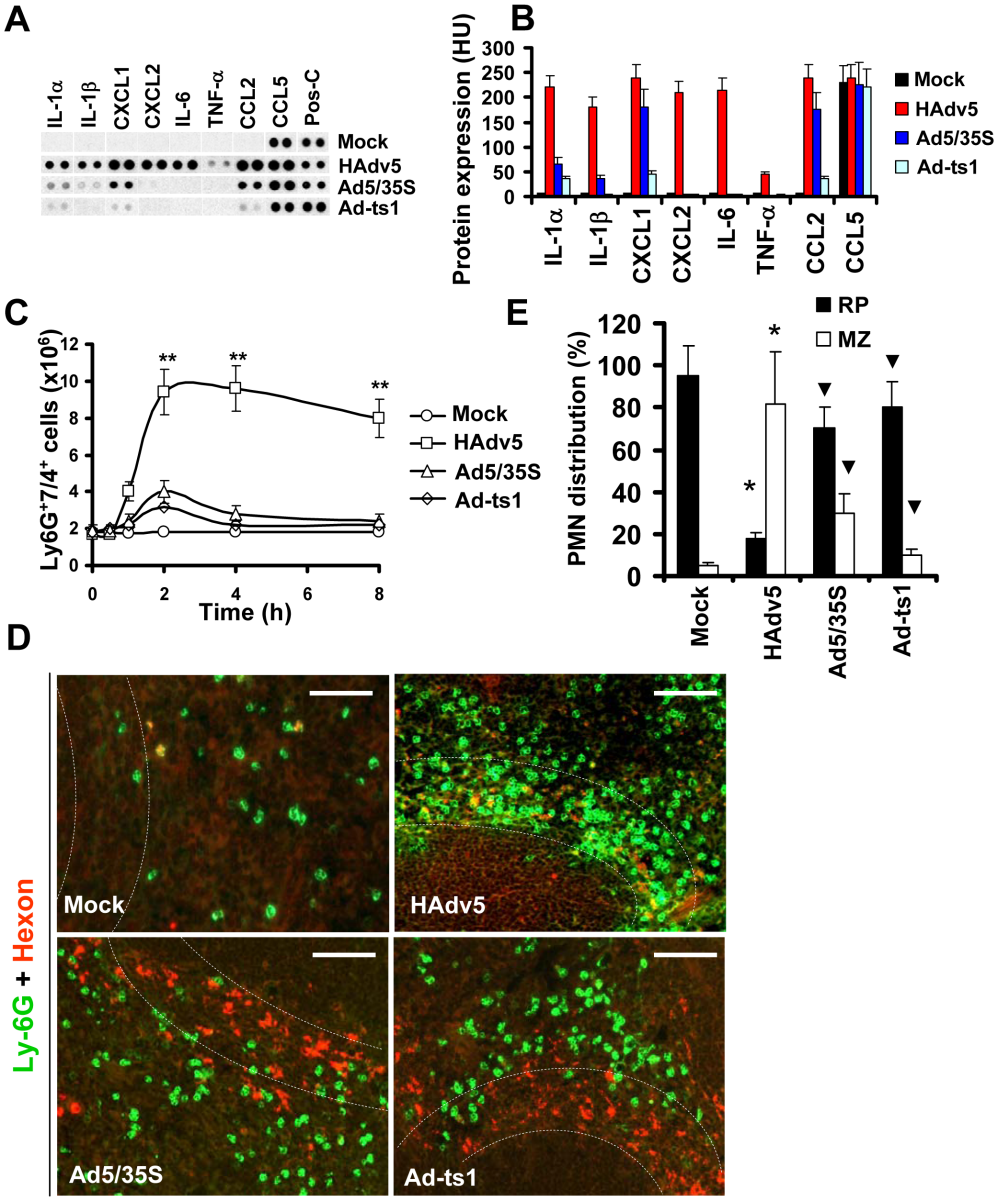
Localization of Ly-6G^+^ cells to the splenic MZ occurs in response to pro-inflammatory stimuli. (A) Mouse cytokine array panel showing differences in the proinflammatory cytokines and chemokines in spleen of wild type mice injected with saline (Mock), Ad5 (HAdv5), Ad5/35S, and Ad-ts1 viruses 1 hour after virus injection, determined by Proteome Profiler antibody array. Representative blots from four independent experiments are shown. Pos-C are dots that show the manufacturer's internal positive control samples on the membrane. Mock – the spleen protein sample of a mouse injected with saline. (B) The amounts of proinflammatory cytokines and chemokines in the spleens of mice 1 hour after Ad injection. HU – histogram units. Graphs show means ± s.d. n = 4. Mock – negative control mice injected with saline. (C) Kinetics of Ly-6G^+^7/4^+^ leukocyte accumulation in the spleen after injection of mice with 10^10^ virus particles of Ad5 (HAdv5), Ad5/35S and Ad-ts1 viruses. The data obtained from three independent experiments is shown. Mock group of mice was injected with phosphate-buffered saline (PBS) only. Splenocytes were harvested, stained with antibodies for Ly-6G and 7/4 cellular markers and analyzed by flow cytometry. N = 4. ** - P<0.01. Error bars represent standard deviation of a mean. (D) Distribution of Ly-6G^+^ leukocytes on sections of the spleens harvested from mice injected 10^10^ virus particles of Ad (HAdv5), Ad5/35S, and Ad-ts1. Mock – mice were administered with saline. The MZ is outlined by the dotted lines. Spleen sections were simultaneously stained with FITC anti-Ly-6G (green) and anti-Ad-hexon (red) antibodies and pictures were taken using a Leica fluorescent microscope. Representative spleen sections for each experimental setting are shown. N = 5. Scale bar is 75 µm (E) Quantitative representation of Ly-6G^+^ (PMN) distribution on sections of the spleens of mice administered with 10^10^ virus particles of indicated viruses 2 hours after virus challenge. The quantification of Ly-6G^+^ cell distribution was done as described in Figure 3B. N = 5. RP – red pulp; MZ – marginal zone. * - P<0.05, compared to corresponding Mock groups. Inverted triangle – P<0.05, compared to Ly-6G^+^ cell distribution observed for corresponding compartments in mice administered with HAdv5. Error bars represent standard deviation of a mean.

Because release of granulocytes from the bone marrow mediated by AMD3100 is non-inflammatory [30], we hypothesized that the recruitment of PMNs in the splenic MZ after Ad administration occurs in response to local inflammatory stimuli released by MZ macrophages that trap virus particles. To test this hypothesis, we analyzed a panel of inflammatory cytokines and chemokines in the spleen after virus administration using a protein-profiler immuno array [10]. Using this method of analysis we earlier showed that the intensity of dots measured in histogram units directly correlates with the absolute amount of the analyte under investigation [31]. This analysis confirmed that the amounts of inflammatory cytokines and chemokines in the spleen were highly elevated after injection of unmodified HAdv5 and not after injection of viruses that are also sequestered by the MZ cells but are known to induce low-level inflammatory cytokine production (Fig. 4A–B). Ad5/35S is an HAdv5-based vector that was previously shown to poorly activate inflammatory cytokine production due to a mutation in the virus fiber protein [32]. The Ad-ts1 is a thermo-sensitive mutant of HAdv2 and cannot escape the endosomal cellular compartment after internalization into the cell [33], [34]. This virus is also known to induce a low-level inflammatory response after intravascular administration [10]. Using flow cytometry analysis, we next found that the number of Ly-6G^+^7/4^+^ cells in the spleen significantly increased only after injection of mice with HAdv5, but not with Ad5/35S or Ad-ts1 viruses (Fig. 4C). Importantly, the distribution of PMNs in the spleens of mice injected with Ad5/35S and Ad-ts1 viruses was significantly different from that observed in mice injected with HAdv5, where the vast majority of Ly-6G^+^ cells was found in the splenic MZ (Fig. 4D–E). In contrast, after mouse injection with Ad5/35 and Ad-ts1 viruses, the majority of PMNs was found in the red pulp and not in the MZ, despite the evident accumulation of virus particles in MZ cells. This distribution of PMNs closely resembles their localization in Mock-injected group, where mice were administered with saline (Fig. 4D–E). Collectively, our analyses showed that in response to intravascular Ad administration, after release from the bone marrow, PMNs enter into and are retained in the splenic MZ. PMNs that are released from the bone marrow via a non-inflammatory stimulus do not enter or become retained within the MZ compartment of the spleen. The Ad5/35S and Ad-ts1 vectors, which induce low-level inflammatory cytokine and chemokine activation, trigger low levels of entry and retention of PMNs in the MZ, despite the efficient accumulation of virus particles in MZ cells (Fig. 4E).

### IL-1α-IL-1RI signaling partially contributes to PMN recruitment into the splenic MZ and requires CXCR2 but not CXCR1 signaling

**Figure 5:**
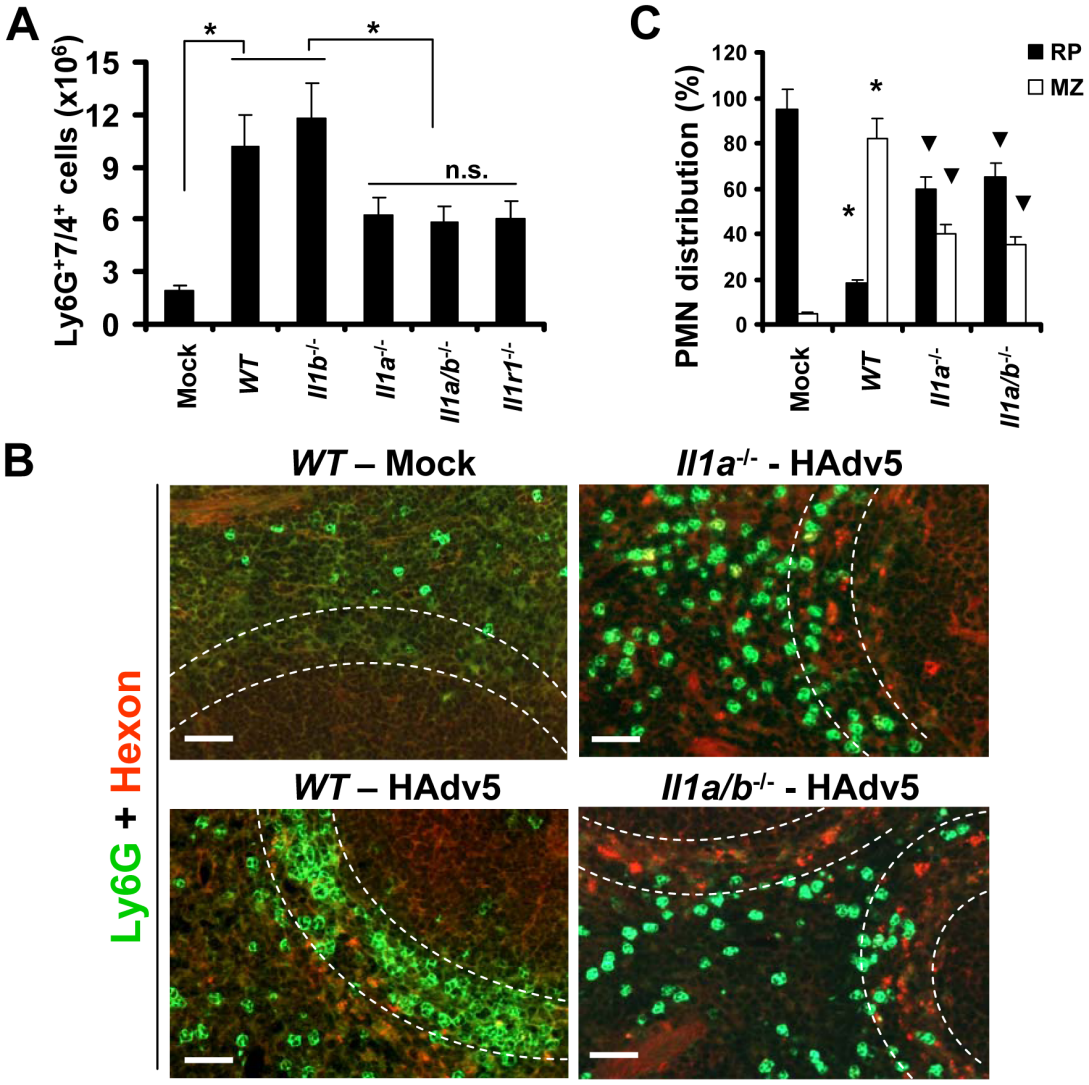
The recruitment of Ly-6G^+^7/4^+^ leukocytes to the splenic marginal zone partially depends on functional IL-1α-IL-1RI signaling, but not on IL-1β. (A) Splenocytes were harvested from *WT*, *Il1a* ^−/−^, *Il1b* ^−/−^, *Il1a/b* ^−/−^, and *Il1r1* ^−/−^ mice injected with Ad 2 hours after the virus challenge. Mock – *WT* mice were injected with saline. N = 5. * - P<0.05. n.s. – not statistically significant, between indicated experimental groups. Error bars represent standard deviation of a mean. (B) Distribution of Ly-6G^+^ leukocytes on sections of the spleens harvested from *WT*, *Il1a* ^−/−^, and *Il1a/b* ^−/−^ mice injected with Ad virus (HAdv5). Mock – mice were injected with saline. The MZ is outlined by the dotted lines. Spleen sections were simultaneously stained with FITC anti-Ly-6G (green) and anti-Ad-hexon (red) antibodies and pictures were taken using a Leica fluorescent microscope. Representative spleen sections for each experimental setting are shown. N = 5. (C) Quantitative representation of Ly-6G^+^ (PMN) distribution on sections of the spleens of mice injected with Ad 2 hours after the virus administration. The quantification of Ly-6G^+^ cell distribution was done as described in Figure 3B. N = 5. RP – red pulp; MZ – marginal zone. * - P<0.05, compared to corresponding mock groups. Inverted triangle – P<0.05, compared to Ly-6G^+^ cell distribution observed for corresponding compartments in *WT* mice.

We previously showed that upon entry into macrophages *in vivo*, Ad activates a stereotypic cascade of inflammatory cytokines and chemokines through activation of IL-1α [10]. IL-1α is a principal pro-inflammatory cytokine produced by the vast majority of cell types in the context of necrotic cell death [35], [36], [37]. Similar to IL-1β, IL-1α binds to IL-1 receptor type 1 (IL-1RI), and both of these cytokines activate identical biological responses downstream of IL-1RI signaling [38], [39], which include inducing CXCL1 and CXCL2 chemokines, which trigger PMN recruitment. To analyze whether IL-1RI signaling or individual IL-1RI ligands were responsible for PMN retention in the splenic MZ, we first administered Ad into wild type (*WT*) mice and mice deficient in IL-1α, IL-1β, IL-1α/β, and IL-1RI, and analyzed the percentage of Ly-6G^+^7/4^+^ cells in the spleen 2 hours after virus administration. This analysis revealed that the number of Ly-6G^+^7/4^+^ cells was significantly higher in the spleens of mice injected with Ad, compared to mock-injected group. However, the number of PMNs in the spleens of mice after Ad injection was two-fold higher in *WT* and *Il1b* ^−/−^ mice, compared to *Il1a* ^−/−^, *Il1a/b* ^−/−^, and *Il1r1* ^−/−^ animals (Fig. 5A). These data suggest that the lack of IL-1α-IL-1RI signaling may either slightly inhibit the egress of PMNs from the bone marrow or selectively prevent their entry into the spleen. Importantly, the analysis of distribution of PMNs between the red pulp and MZ revealed that while in *WT* mice, over 80% of PMNs localized within the MZ compartment, in both *Il1a* ^−/−^ and *Il1a/b* ^−/−^ animals, the PMN distribution was significantly different from that observed in both the virus-injected and saline-injected *WT* mice. Specifically, in *Il1a* ^−/−^ and *Il1a/b* ^−/−^ mice, on average, 60% of cells localized to red pulp and 40% localized to the MZ (Fig. 5B–C). Although the proportion of PMNs localizing to the MZ in *Il1a* ^−/−^ and *Il1a/b* ^−/−^ animals was significantly reduced compared to the Ad-injected *WT* mice, this number was still significantly higher, compared to that observed in saline-injected animal where only 5–7% of PMNs were found in the splenic MZ. These data indicate that IL-1α-IL-1RI signaling is only partially responsible for guiding the recruitment of PMNs into the splenic MZ after Ad injection.

**Figure 6:**
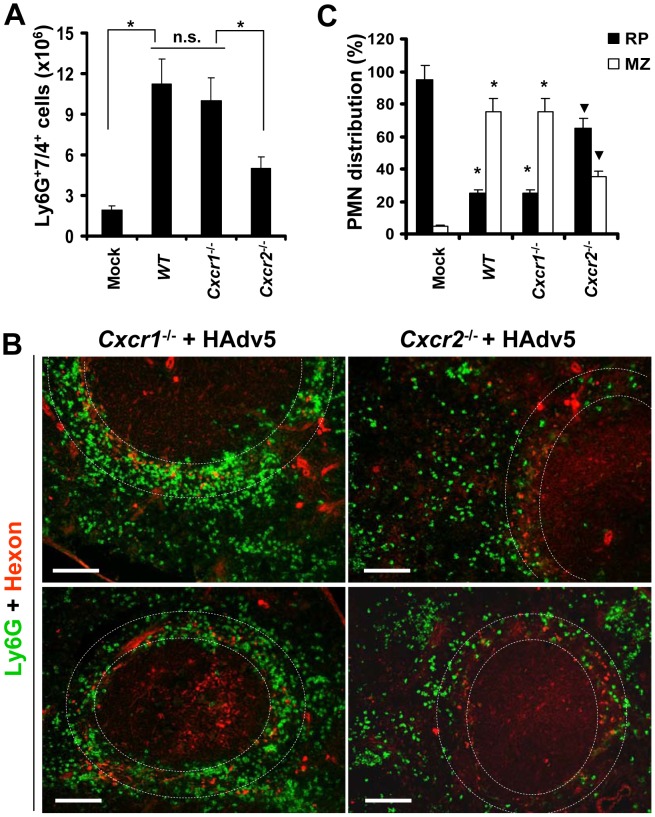
The retention of Ly-6G^+^ cells in the splenic marginal zone requires CXCR2, but not CXCR1, signaling. (A) Splenocytes were harvested from *WT*, *Cxcr1* ^−/−^, and *Cxcr2* ^−/−^ mice injected with Ad 2 hours after the virus challenge. Mock – *WT* mice were injected with saline. N = 5. * - P<0.05. n.s. – not statistically significant, between indicated experimental groups. Error bars represent standard deviation of a mean. (B) Distribution of Ly-6G^+^ leukocytes on sections of the spleens harvested from *Cxcr1* ^−/−^ and *Cxcr2* ^−/−^ mice injected with Ad virus (HAdv5). Spleen sections were simultaneously stained with FITC anti-Ly-6G (green) and anti-Ad-hexon (red) antibodies and pictures were taken using a Leica fluorescent microscope. Representative spleen sections for each experimental setting are shown. N = 5. Scale bar is 150 µm. (C) Quantitative representation of Ly-6G^+^ (PMN) distribution on sections of the spleens of indicated gene deficient mice injected with Ad 2 hours after the virus administration. The quantification of Ly-6G^+^ cell distribution was done as described in Figure 3B. N = 5. RP – red pulp; MZ – marginal zone. * - P<0.05, compared to corresponding mock groups. Inverted triangle – P<0.05, compared to Ly-6G^+^ cell distribution observed for corresponding compartments in *WT* mice.

PMN recruitment to the sites of infection occurs in response to chemotactic stimuli, with CXCL1 and CXCL2 being among the most potent chemokines promoting PMN migration [19]. However, both of these chemokines can bind to CXCR1 and CXCR2 receptors on neutrophils to promote their migration in various pathological conditions [19], [40], [41]. To further delineate which chemokine receptor plays a role in guiding PMN recruitment into the splenic MZ after intravascular Ad injection, we administered virus to *WT* and *Cxcr1* ^−/−^ or *Cxcr2* ^−/−^ mice. The analysis of the percentages of Ly-6G^+^7/4^+^ cells in the spleen showed that in both *WT* and *Cxcr1* ^−/−^ mice, the numbers of PMNs in the spleen were identical 2 hours after the virus injection. Although the number of PMNs in the spleens of *Cxcr2* ^−/−^ mice was significantly higher than found in saline-injected *WT* animals (Mock group, Fig. 6A), it was two-fold lower, compared to the percentage of PMNs recovered from the spleens of virus-injected *WT* and *Cxcr1* ^−/−^ animals. The analysis of PMN distribution between the red pulp and MZ after injection of mice with Ad further showed that the distribution of Ly-6G^+^ cells in splenic parenchyma was identical in *WT* and *Cxcr1* ^−/−^ mice, with over 80% of cells localizing in the splenic MZ in both variants (Fig. 6B–C). In contrast, in *Cxcr2* ^−/−^ mice, only 30–35% of PMNs localized to the splenic MZ, and the majority of Ly-6G^+^ cells localized to red pulp after Ad administration. These data demonstrate that efficient PMN recruitment and/or retention in the splenic MZ requires functional CXCR2, and not CXCR1, signaling. It is also consistent with, and qualitatively and quantitatively phenocopies, the PMN distribution in *Il1a* ^−/−^, *Il1a/b* ^−/−^, and *Il1r1* ^−/−^ mice, and suggests that CXCL1 and CXCL2 chemokines (Fig. 4A), which are activated downstream of IL-1α-IL-1RI signaling in response to intravenous Ad injection, signal through the CXCR2 receptor on PMNs to mediate their recruitment and retention in the splenic MZ, the anatomic compartment that contains macrophage populations sequestering Ad from the blood.

### MARCO^+^ macrophages are eliminated from the splenic MZ after interaction with Ad

**Figure 7:**
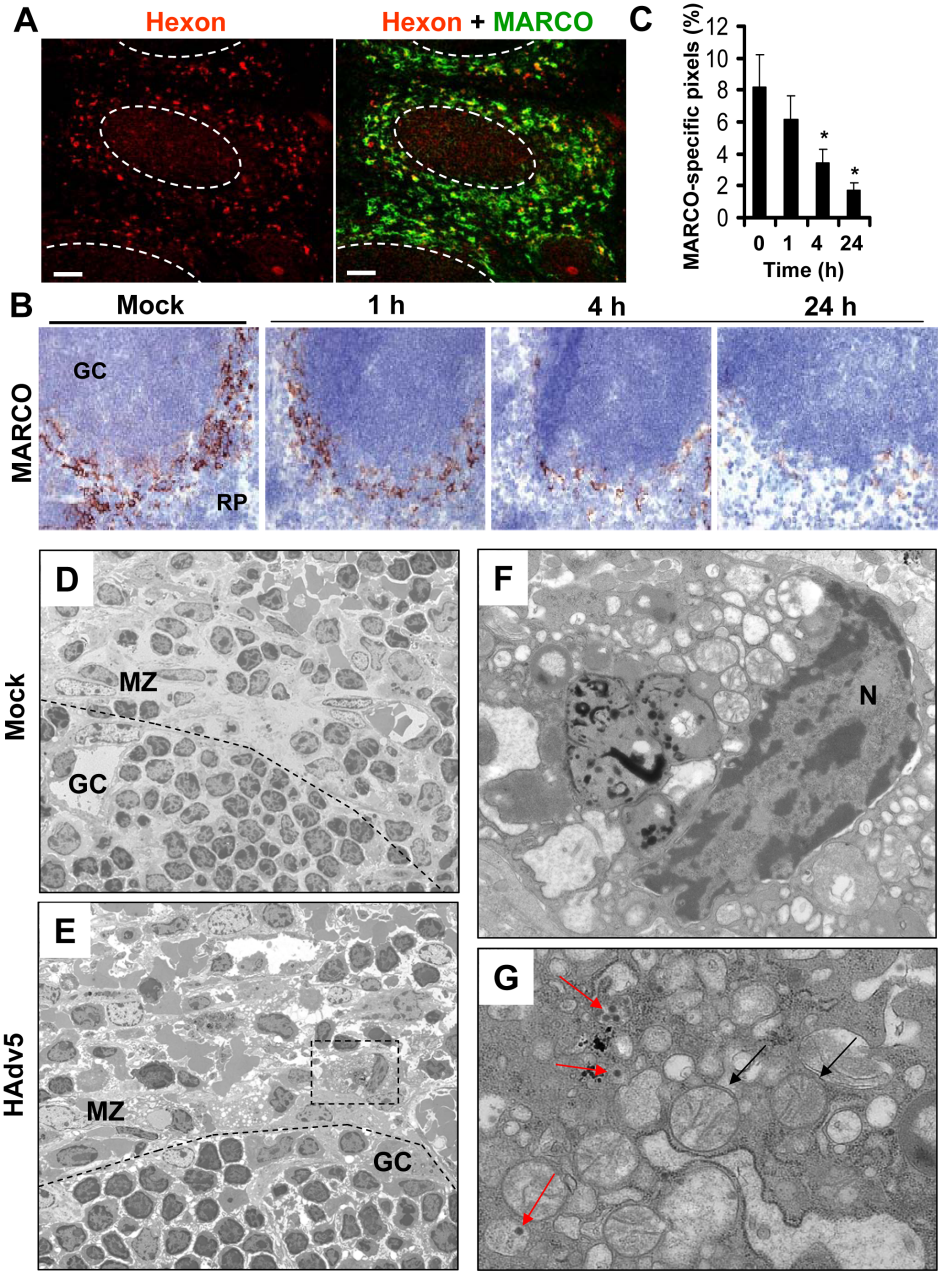
MARCO^+^ marginal zone cells are eliminated from the spleen after sequestering Ad from the blood. (A) Distribution of Ad particles (stained with anti-Hexon Ab, red) and MACRO^+^ cells (stained with anti-MARCO Ab, green) on spleen sections 1 hour after intravascular virus injection. Co-localization of virus particles with MARCO^+^ staining appears as yellow. The anatomic borders of splenic germinal centers are depicted with dotted lines. Scale bar is 50 µm. Representative images are shown. N = 25. (B) Immunohistochemical analysis of MARCO^+^ marginal zone macrophages on sections of spleen at different times after Ad administration. Spleens of mice injected with the virus were harvested at indicated times and stained with MARCO-specific antibodies. Sections were counter-stained with hematoxylin to visualize splenic anatomical compartments. Mock – spleen sections were prepared from mice injected with saline only. GS – germinal center. RP – red pulp. Representative fields are shown. N = 5. (C) Quantitative representation of MARCO^+^-specific staining on splenic sections of mice after Ad administration. N = 4 per experimental group per time point. Marker-specific pixels (brown staining) were quantified using MetaMorph software on low-power images of spleen sections with average distribution of MARCO-specific staining collected from three sections and at three depth levels. * - P<0.05. Error bars represent standard deviation of mean. (D–G) Transmission electron microscopy analysis of ultra-thin sections of spleens harvested from saline-injected mice (D) or Ad-injected mice (E) 4 hours after the virus injection. MZ – marginal zone; GC – germinal center. The anatomic borders of germinal centers are depicted with a punctuated line. (F and G) Punctuated rectangle depicts a marginal zone macrophage that contains Ad particles (red arrows on G) and morphologically distorted mitochondria (F and black arrows on G). N – nucleus. Representative images of virus-containing cells in the marginal zone are shown.

After intravascular administration, Ad particles are trapped in the residential MARCO^+^ macrophages in the splenic MZ (Fig. 7A and [10]). We next analyzed the kinetics of changes in the MARCO^+^ cell population in the spleen after virus administration over time. Using immuno-histochemical staining of spleen sections with anti-MARCO-specific antibodies, we found that the MARCO^+^ MZ cells progressively disappear, and by 24 hours after virus injection, the cellularity of MZ was evidently reduced, compared to control, saline-injected groups (Fig. 7B–C). The disappearance of MARCO^+^ cells from the splenic MZ was dose-dependent and occurred at virus doses of 10^10^ per mouse and higher (Figure S1). To distinguish between the possibilities that MARCO^+^ cells might have migrated out of the splenic MZ to other host compartments or died *in situ*, similarly to CD68^+^ residential macrophages in the liver [42], we conducted an evaluation of ultra-structural changes within the MZ cells that contain Ad particles using conventional transmission electron microscopy. This analysis revealed that unlike MZ macrophages in saline-injected animals, virus-containing MZ macrophages in Ad-injected mice were filled with abnormal vacuolized cytosolic compartments and grossly-distorted mitochondria (Fig. 7D–G). Therefore, the virus-containing cells in the splenic MZ are unlikely to maintain their functional physiological state, but instead are undergoing a catastrophic disorganization of the cytosolic compartments that is consistent with initiation and/or execution of a cell death program *in situ*.

**Figure 8:**
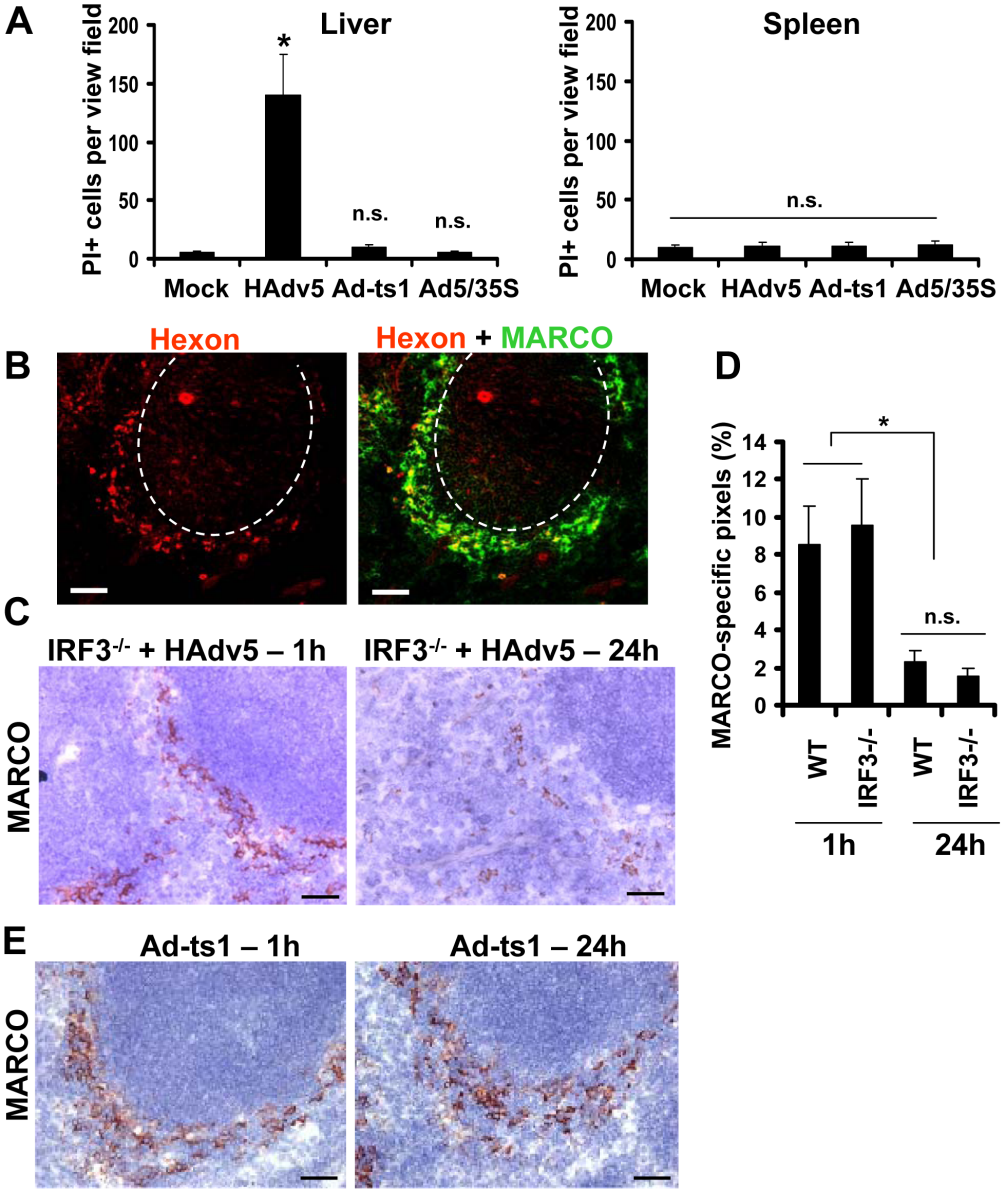
MARCO^+^ cells are eliminated from the splenic MZ through an IRF3-mediated necrosis-independent pathway. (A) Quantification of propidium iodide (PI)-positive cells on unprocessed sections of livers and spleens of mice injected with 10^10^ virus particles of Ad (HAdv5), Ad-ts1, or Ad5/35S viruses 1 hour after the virus challenge. Mice were injected with indicated viruses intravenously and 1 hour later, mice were administered with PI and sacrificed 5 minutes later. Spleens and livers were harvested for histological analyses. Four sections of the spleen at three depth levels were prepared for each mouse and the number of PI-positive cells per low-power view-field was quantified. N = 5. * - P<0.05. n.s. – not significant. Mock – control group of mice injected with saline. (B) Distribution of Ad particles (stained with anti-Hexon Ab, red) and MACRO^+^ cells (stained with anti-MARCO Ab, green) on spleen sections of *Irf3* ^−/−^ mice 1 hour after intravascular virus injection. Co-localization of virus particles with MARCO^+^ staining appears as yellow. The anatomic borders of splenic germinal centers are depicted with dotted lines. Scale bar is 50 µm. Representative images are shown. N = 8. (C) Immunohistochemical analysis of MARCO^+^ marginal zone macrophages on sections of spleens of *Irf3* ^−/−^ mice at different times after Ad administration. Spleens of *Irf3* ^−/−^ mice injected with the virus were harvested at indicated times and stained with MARCO-specific antibodies. Sections were counter-stained with hematoxylin to visualize splenic anatomical compartments. Representative fields are shown. N = 5. Scale bar is 40 µm (D) Quantitative representation of MARCO^+^-specific staining on splenic sections of *WT* and *Irf3* ^−/−^ mice after Ad administration. N = 4 per experimental group per time point. Marker-specific pixels (brown staining) were quantified using MetaMorph software on low-power images of spleen sections with average distribution of MARCO-specific staining collected from three sections and at three depth levels. * - P<0.05. Error bars represent standard deviation of mean. (E) Immunohistochemical analysis of MARCO^+^ marginal zone macrophages on sections of spleens of *WT* mice at 1 hour and 24 hours after administration of Ad-ts1 virus. Spleens of mice injected with Ad-ts1 virus were harvested at indicated times and stained with MARCO-specific antibodies. Sections were counter-stained with hematoxylin to visualize splenic anatomical compartments. Representative fields are shown. N = 5. Scale bar is 40 µm.

Depending on physiological or patho-physiological stimuli, cells can initiate and execute various cell demise programs [43], [44]. We recently found that upon sequestering Ad particles from the blood, CD68^+^ residential macrophages in the liver (Kupffer cells) initiate and execute a unique necrotic-type cell death pathway that depends on the transcription factor IRF3 [42]. This type of cell death was associated with the major disorganization of the cytosolic compartments and induction of plasma membrane permeability to propidium iodide (PI), which can be detected *in situ* via analyzing PI-stained nuclei in the livers of virus-injected mice [42]. To define whether MARCO^+^ macrophages in the spleen also undergo necrotic-type cell death after interaction with Ad, we administered Ad and PI to mice and analyzed the distribution of PI^+^ cells in sections of liver and spleen 1 hour after virus injection. Consistent with previous observations, this analysis showed that after injection of mice with Ad, the liver parenchyma contained up to 150 PI^+^ cells per view field. However, we failed to observe PI^+^ cells in the spleen at the same time point (Fig. 8A), suggesting that MZ macrophages do not undergo necrotic-type cell death after interaction with Ad. In agreement with earlier studies [42], [45], we further found that the Ad-ts1 and Ad5/35S virus vectors (that induce inflammatory response poorly (Fig. 4)), do not trigger PI plasma membrane permeability in the spleen or liver (Fig. 8A).

To definitively exclude that MZ macrophages undergo IRF3-dependent necrosis after interaction with adenovirus, we administered Ad into *WT* and *Irf3* ^−/−^ mice and analyzed persistence of the MARCO^+^ cell population over time. This analysis showed that at 1 hour after the virus injection, MARCO^+^ macrophages efficiently trapped virus particles from the blood (Fig. 8B). However, by 24 hours after virus injection, MARCO^+^ cells were missing in both the *WT* and *Irf3* ^−/−^ mice (Fig. 8C–D). In contrast, administration of *WT* mice with Ad-ts1 virus showed that this macrophage population remained in the MZ 24 hours after the virus injection irrespective of the efficient accumulation of virus particles in MZ cells (Fig. 4E). Collectively, this data shows that resident macrophages in the liver and spleen respond to Ad particles via distinct pathways. Although after sequestering Ad particles from the blood, both liver Kupffer cells [42], [45], [46] and MZ macrophages undergo catastrophic disorganization of cytosolic compartments, MZ macrophages do not lose their plasma membrane integrity and are eliminated from the spleen in both *WT* and *Irf3* ^−/−^ mice.

### Complement cooperates with the IL-1α-IL-1RI-CXCR2 signaling axis to recruit PMNs to the splenic MZ and eliminate virus-containing cells

**Figure 9:**
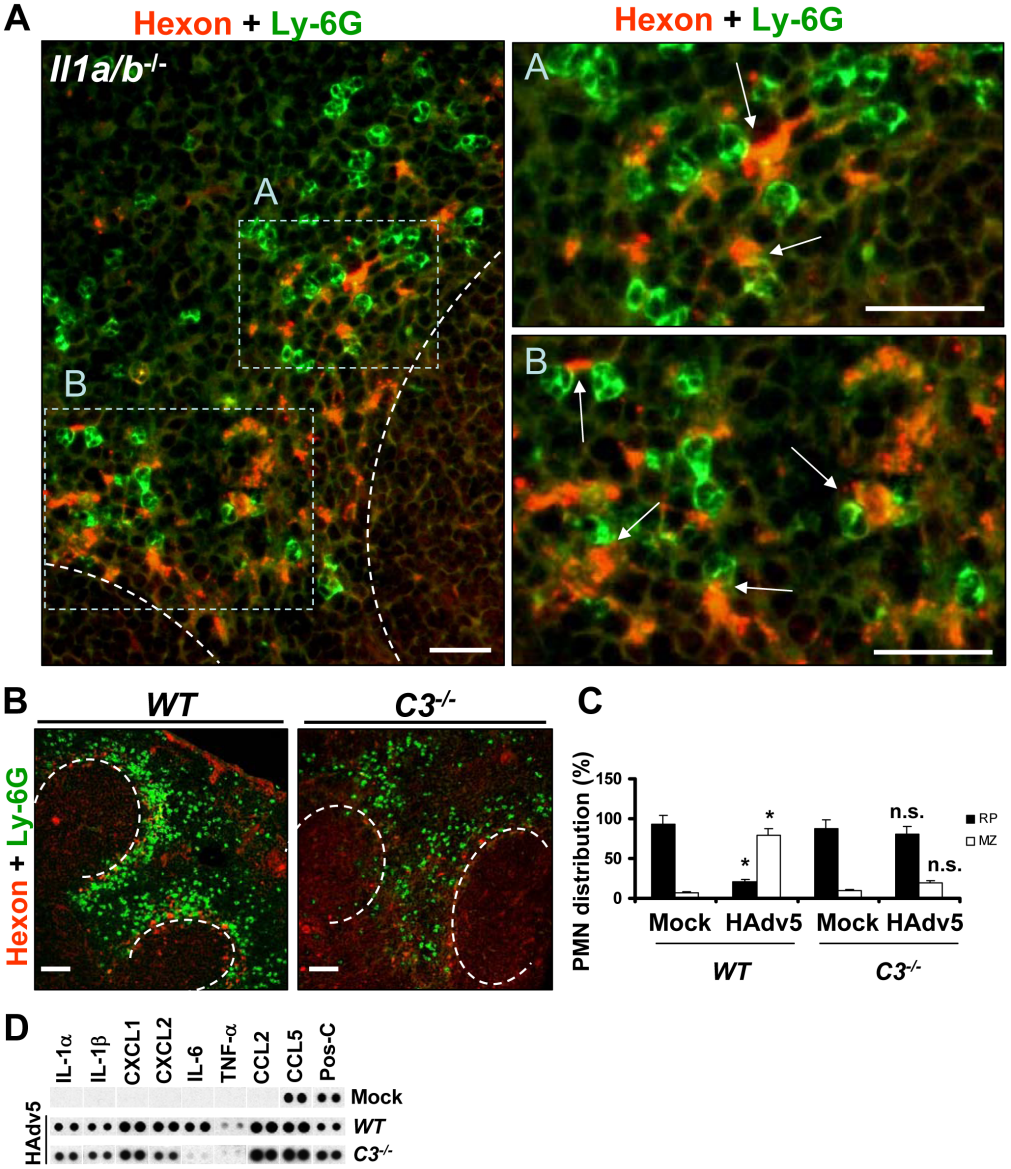
Complement component C3 is required for recruitment and retention of Ly-6G^+^ cells in the splenic MZ after Ad injection. (A) Localization of Ly-6G^+^ cells in the MZ of *Il1a/b* ^−/−^ mice 2 hours after Ad injection in relation to virus-containing MZ cells. Spleen sections of virus-injected *Il1a/b* ^−/−^ mice were simultaneously stained with FITC anti-Ly-6G (green) and anti-Ad-hexon (red) antibodies and pictures were taken using a Leica fluorescent microscope. The regions of the spleen that are selected by rectangles A and B on the left panel are shown on the right panels at higher magnification. Scale bar is 25 µm. Anatomical borders of germinal centers are depicted by the dotted lines. Ly-6G^+^ cells that are in contact with virus-containing cells in the MZ are indicated by arrows. Representative spleen sections are shown. N = 5. (B) Distribution of Ly-6G^+^ leukocytes on sections of the spleens harvested from *WT* and complement component C3-deficient (*C3* ^−/−^) mice injected with Ad virus. Spleen sections were simultaneously stained with FITC anti-Ly-6G (green) and anti-Ad-hexon (red) antibodies and pictures were taken using a Leica fluorescent microscope. Representative spleen sections for each experimental setting are shown. N = 5. Scale bar is 100 µm. (C) Quantitative representation of Ly-6G^+^ (PMN) distribution on sections of the spleens of *WT* and *C3* ^−/−^ mice injected with Ad 2 hours after the virus administration. The quantification of Ly-6G^+^ cell distribution was done as described in Figure 3B. N = 5. RP – red pulp; MZ – marginal zone. * - P<0.05, compared to corresponding mock groups. Inverted triangle – P<0.05, compared to Ly-6G^+^ cell distribution observed for corresponding compartments in *WT* mice. (D) Mouse cytokine array panel showing expression of the proinflammatory cytokines and chemokines in spleens of *WT* and *C3* ^−/−^ mice injected with saline (Mock) or Ad5 (HAdv5) 1 hour after virus injection, determined by Proteome Profiler antibody array. Representative blots from three independent experiments are shown. Pos-C are dots that show the manufacturer's internal positive control samples on the membrane.

The finding that MZ macrophages do not lose plasma membrane integrity after sequestering Ad from the blood was surprising and reveals a clear divergence in macrophage responses to Ad in a tissue-specific manner. Furthermore, although after intravascular Ad injection PMNs do enter the liver [21], [22], they are unlikely to directly contribute to elimination of virus-containing Kupffer cells, since these cells undergo IRF3-dependent necrosis in a cell-autonomous fashion [42]. In contrast, the recruitment and retention of PMNs in the splenic MZ, that is the natural host compartment for virus-trapping MARCO^+^ macrophages, may indicate that these PMNs have a functional role in eliminating virus-containing cells from the MZ. To investigate this possibility, we first analyzed the localization of Ly-6G^+^ cells in the splenic MZ of *Il1a/b* ^−/−^ mice in relation to localization of the Ad-containing cells. This analysis revealed that when IL-1-IL-1RI signaling is lacking, those PMNs that enter and are retained in the MZ localize in the immediate proximity to the virus-containing cells (Fig. 9A). This characteristic PMN localization in the immediate proximity to the virus-containing MZ cells suggests that even without IL-1R ligands, there are additional, locally-presented chemotactic stimuli that guide PMN migration to the MZ and position them in contact with Ad-containing cells. Besides CXC chemokines, CXCL1 and CXCL2, the activated components of a complement cascade, e.g. C3a and C5a, were shown to be potent chemoattractants for PMN localization to the sites of infection [40]. Furthermore, extensive earlier studies showed that complement is indeed activated in response to intravascular Ad administration *in vivo* and C3a fragments are readily detectable in the blood of virus-injected mice [47]. Of note, the Ad-ts1 virus that fails to initiate IL-1RI-dependent chemokine activation ([10] and Fig. 4A), PMN recruitment to the MZ (Fig. 4E), and MARCO^+^ cell death (Fig. 8E), also failed to trigger complement activation *in vivo* [47]. To analyze whether complement may play a role in PMN recruitment to the splenic MZ after Ad injection, we administered the virus to *WT* and complement component C3-deficient (*C3* ^−/−^) mice. The analysis of PMN distribution on the spleen sections of virus-injected mice showed that the proportion of PMNs that localized to the MZ after Ad injection in *C3* ^−/−^ mice was significantly lower than in *WT* mice (Fig. 9B–C). While over 80% of PMNs localized to the splenic MZ in *WT* mice, only 22% of PMNs localized to the MZ in *C3* ^−/−^ mice. Accordingly, the majority of PMNs in virus-injected *C3* ^−/−^ mice were distributed throughout the red pulp of the spleen. We further confirmed that *C3* ^−/−^ mice were not deficient in initiation of IL-1α-IL-1RI signaling and production of CXCL1 and CXCL2 chemokines (Fig. 9D). We observed a reduced expression of IL-6 in *C3* ^−/−^ mice after virus injection as compared to *WT* mice. However, the *Il6* ^−/−^ mice exhibited no defects in activating IL-1α-IL-1RI-signaling and CXCL1 and CXCL2 chemokine expression or recruiting PMNs to the splenic MZ after virus administration (data not shown).

**Figure 10:**
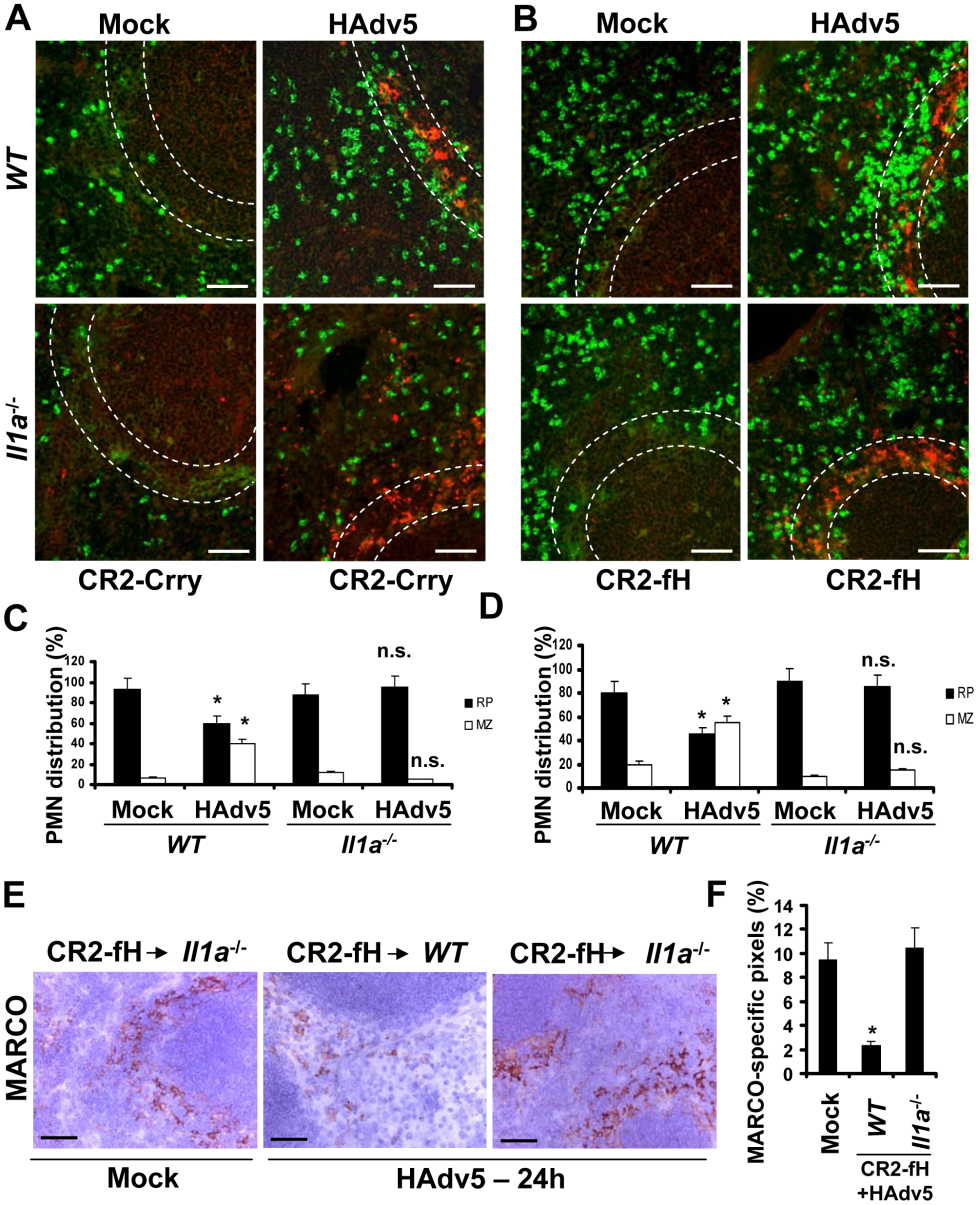
IL-1α-dependent signaling and complement cooperate in promoting the recruitment of Ly-6G^+^ leukocytes that eliminate MARCO^+^ cells from the splenic MZ. (A) Distribution of Ly-6G^+^ leukocytes on sections of the spleens harvested from *WT* and *Il1a* ^−/−^ mice pre-treated with complement activation inhibitor CR2-Crry (50 µg/mouse) 1 hour prior to Ad injection. Spleen sections were simultaneously stained with FITC anti-Ly-6G (green) and anti-Ad-hexon (red) antibodies and pictures were taken using a Leica fluorescent microscope. Representative spleen sections for each experimental setting are shown. N = 5. Scale bar is 50 µm. (B) Distribution of Ly-6G^+^ leukocytes on sections of the spleens harvested from *WT* and *Il1a* ^−/−^ mice pre-treated with alternative complement activation pathway inhibitor CR2-fH (50 µg/mouse) 1 hour prior to Ad injection. Spleen sections were simultaneously stained with FITC anti-Ly-6G (green) and anti-Ad-hexon (red) antibodies and pictures were taken using a Leica fluorescent microscope. Representative spleen sections for each experimental setting are shown. N = 5. Scale bar is 50 µm. (C–D) Quantitative representation of Ly-6G^+^ (PMN) distribution on sections of the spleens of *WT* and *Il1a* ^−/−^ mice pre-treated with CR2-Crry (C) or CR2-fH (D) complement inhibitors 1 hour prior to Ad administration. The quantification of Ly-6G^+^ cell distribution was done as described in Figure 3B. N = 5. RP – red pulp; MZ – marginal zone. * - P<0.05, n.s.- not significant compared to corresponding mock (saline-injected) groups. (E) Immunohistochemical analysis of MARCO^+^ marginal zone macrophages on sections of spleens of mock-injected or Ad-injected (HAdv5) *Il1a* ^−/−^ mice pre-treated with CR2-fH 1 hour prior to Ad administration. The scale bar is 40 µm. Representative pictures are shown. N = 5. (F) Quantitative representation of MARCO^+^-specific staining on splenic sections of *WT* and *Il1a* ^−/−^ mice pre-treated with 50 µg of CR2-fH complement inhibitor prior to Ad administration (HAdv5). N = 4 per experimental group per time point. Marker-specific pixels (brown staining) were quantified using MetaMorph software on low-power images of spleen sections with average distribution of MARCO-specific staining collected from three sections and at three depth levels. * - P<0.05. Error bars represent standard deviation of mean.

To analyze whether IL-1α-IL-1RI signaling and complement cooperate in PMN recruitment to the splenic MZ after intravascular Ad injection, we utilized a pharmacological approach of blocking all or only alternative complement activation pathways by pre-treating mice with CR2-Crry or CR2-fH complement inhibitory proteins [48] prior to Ad administration. Pre-treatment of *WT* mice with CR2-Crry [49] or CR2-fH [50] prior to Ad injection resulted in partial re-distribution of PMNs away from the MZ (Fig. 10A–D, *WT* panels). However, pre-treatment of IL-1α-deficient mice with complement-blocking proteins resulted in complete inhibition of PMN localization to the MZ, despite evident accumulation of virus particles in MZ cells (Fig. 10A–D, *Il1a* ^−/−^ panels). The analysis of MARCO^+^ cell populations in *WT* and *Il1a* ^−/−^ mice after Ad injection with pre-treatment of mice with CR2-Crry (not shown) or CR2-fH showed that the pre-treatment of *WT* mice with CR2-fH prior to Ad injection did not prevent elimination of MARCO^+^ cells from the MZ 24 hours after the virus administration. In contrast, pre-treatment of mice deficient in IL-1α with CR2-fH prior to the virus administration resulted not only in preservation of MARCO^+^ cells in the MZ, but also in appearance of MARCO^+^ cells in the red pulp – a response that was not observed in CR2-fH-treated *WT* mice (Fig. 10E–F). Taken together, these analyses showed that the recruitment of PMNs to the MZ after Ad injection is deficient in complement component *C3* ^−/−^ mice. They also showed that complement and IL-1α-dependent signaling cooperate in PMN recruitment into the MZ. Pharmacological inhibition of complement in IL-1α-deficient mice prevented PMN accumulation in the splenic MZ and elimination of MARCO^+^ virus-containing cells.

## Discussion

Adenovirus has been adopted as a highly efficient gene transfer vehicle to deliver exogenous genes to various cell types and tissues *in vivo*. However, when the virus is injected intravenously, it induces acute and potent innate immune and inflammatory responses that can be lethal to the host [3], [4], [5]. Although numerous events, including cytokine and chemokine gene activation [17], [18], complement activation [51], [52], release of platelet activating factor [53], and Kupffer cell death [42], [46] were shown to ensue upon intravascular Ad administration, the specific contribution of each of these factors in activating systemic host inflammatory responses and their functional consequences remain poorly defined. Furthermore, the vast majority of studies conducted to date were focused largely on defining the most prominent *molecular* mediators that lead to acute systemic Ad-induced toxicity. Using a panel of genetic mouse models that are deficient in key mediators of innate immunity and inflammation, we previously found that Ad induces activation of a stereotypic pro-inflammatory cytokine and chemokine cascade through the IL-1α-IL-1RI signaling pathway [10]. However, the relevance of this pathway to the induction of *cellular* innate responses remains unclear. Here we analyzed host cellular inflammatory response to Ad in wild type mice and mice deficient in IL-1α-IL-1RI signaling pathway genes, CXCR1 and CXCR2 chemokine receptors, and complement. Our studies revealed that intravascular Ad administration leads to a release of polymorphonuclear leukocytes with pro-inflammatory Ly-6G and 7/4 cellular markers from the bone marrow into the blood and their entry and retention in the peripheral tissues, such as spleen.

Previous studies showed that in response to intravascular Ad injection, no leukocyte retention was seen in the sinusoids of the liver [21]. In contrast to this observation, we found that PMN recruitment and retention to the spleen was a highly coordinated process that leads to redistribution of recruited cells to a confined and specific anatomical compartment of the spleen, the marginal zone. This area of the spleen is occupied by MZ macrophages that express the MARCO cell surface marker and selectively trap Ad particles after intravascular virus administration [10]. Trafficking of blood-borne material and cells through the spleen is highly complex and the exact routes of blood flow through the splenic parenchyma remain a debatable issue [23]. To define the exact natural trafficking route of Ly-6G^+^7/4^+^ PMNs through the spleen without challenging animals with Ad, we injected mice with the small molecule drug AMD3100. Injection of mice with AMD3100 did not induce inflammatory cytokines and chemokine in the spleen (data not shown) and over 95% of Ly-6G^+^ PMNs were found in the splenic red pulp, outside of the MZ (Fig. 3). In contrast, after Ad administration, Ly-6G^+^ PMNs localized virtually exclusively to the splenic MZ, indicating the existence of specific and local stimuli that trigger PMN recruitment and retention in this defined anatomical compartment.

CXC family chemokines, CXCL1 and CXCL2, are among the most potent known chemoattractants that promote neutrophil migration and activation [19]. In response to intravascular Ad injection, both CXCL1 and CXCL2 are produced in the spleen downstream of IL-1α-IL-1RI signaling, a process that is initiated by MZ macrophages that trap Ad particles from the blood [10]. Here we found that in mice deficient in IL-1α, IL-1α/β, or IL-1RI, the recruitment of PMNs to the splenic MZ is reduced only partially, suggesting that additional stimuli may exist that guide PMN migration to and/or retention in the MZ. We also found that this phenotype was reproduced in CXCR2-deficient mice, but not in mice lacking CXCR1. We further serendipitously found that in mice lacking complement component C3, the recruitment of PMNs to the MZ after Ad injection was greatly reduced compared to the *WT* mice. Although complement is known to be activated after intravascular Ad delivery, the role of complement in driving neutrophil recruitment to the compartments in peripheral tissues that house virus-containing cells has never been demonstrated. Importantly, pharmacological blocking of complement activation in IL-1α-deficient, but not in *WT*, mice completely prevented PMN recruitment into the MZ despite the efficient trapping of virus particles by MZ cells. These data indicate that IL-1α-dependent signaling and complement cooperate in PMN recruitment to the splenic MZ and positioning of these cells in proximity to virus-containing macrophages.

Neutrophils are among the most abundant professional phagocytic cells of the myeloid lineage and, together with circulating monocytes and tissue macrophages, form the effector arm of innate immunity [41]. Although both PMNs and macrophages can efficiently engulf and destroy extracellular pathogens, their effector mechanisms are distinct and non-redundant. Furthermore, macrophages and PMNs are known to cooperate at mounting host protective immune responses to both intracellular and extracellular bacterial pathogens [41] via diverse mechanisms. Phagocytosis, degranulation, and release of nuclear DNA fragments (NETs) are thought to be the principal PMN effector mechanisms enabling killing of pathogens [19]. However, it was also reported that the cooperation between macrophages (that may harbor the pathogens) and PMNs can occur through the transfer of anti-microbial molecules from PMNs to macrophages during degranulation of activated neutrophils [54], [55]. While components of neutrophilic granules can kill bacterial pathogens, they also can exert cytotoxic activity and cause rapid death of surrounding cells [56]. In this context, we found that the physiological consequence of the PMN migration and retention in the splenic MZ and their localization in proximity to MARCO^+^ macrophages is elimination of the virus-containing cells from the MZ. The Ad-*ts1* virus, which causes no PMN retention in the MZ, failed to trigger elimination of virus-containing MARCO^+^ cells. Furthermore, pharmacological inhibition of complement in IL-1α-deficient mice resulted in re-distribution of PMNs from the MZ to red pulp and preservation of the MARCO^+^ cell population despite effective retention of virus particles. It is noteworthy that in our recent study we found that *Tlr4* ^−/−^ mice also exhibited reduced PMN recruitment into the splenic MZ and failed to clear the virus from the spleen, when analyzed for the amounts of Ad DNA present 24 hours after virus infection by real-time PCR ([57], Figs. S15–S16).

**Figure 11:**
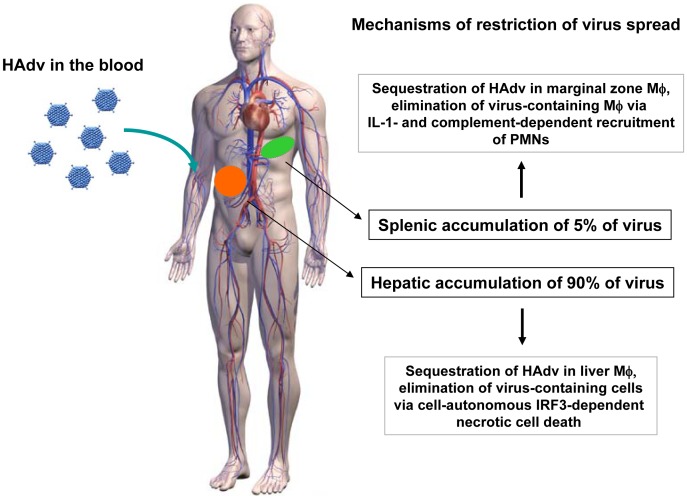
The model of distinct molecular mechanisms engaged by tissue residential macrophages in the liver and spleen to limit systemic spread of adenovirus. After intravascular adenovirus administration, macrophages in the liver and spleen sequester virus particles from the blood. In the liver, tissue residential macrophages, Kupffer cells, undergo cell-autonomous IRF3-dependent necrosis. In the spleen, MARCO^+^ marginal zone macrophages activate IL-1α-IL-1RI-signaling and complement that recruit PMNs, which eliminate virus-containing cells. In both tissues, the functional consequence of host response to Ad is elimination of virus-containing cells and restriction of systemic virus spread.

Ad administration via an intravascular route causes multifaceted systemic innate immune and inflammatory responses. Numerous comprehensive analyses of virus bio-distribution after intravascular injection have shown that the liver and spleen accumulate over 90 percent of the administered virus particles [58], [59], [60], [61], [62]. Our study revealed that although the functional consequence of Ad interaction with tissue macrophages is virus elimination in both the liver and spleen, the molecular mechanisms employed by the host to restrict systemic virus spread are distinctly different (Fig. 11). While liver residential macrophages, Kupffer cells, undergo cell-autonomous IRF3-dependent necrosis after trapping Ad particles from the blood [42], MARCO^+^ MZ macrophages in the spleen do not undergo cell-autonomous necrosis, but rather activate IL-1α-IL1RI-dependent pro-inflammmatory cytokine and chemokine production [10]. The interaction of Ad with splenic macrophages also triggers complement activation [47] and requires complement component C3, which cooperates with CXCL1 and CXCL2 chemokines to recruit and retain PMNs with a pro-inflammatory phenotype (Ly-6G^+^/7/4^+^) into the splenic MZ in contact with virus-containing cells. Activated PMNs degranulate locally and damage both the virus and virus-containing cells, leading to their demise and elimination from the spleen. Collectively, cooperation of tissue macrophages, which sequester Ad from the blood, and PMNs results in effective reduction of the virus load in the spleen. This model provides further support to the concept of molecular and functional specialization of tissue residential macrophages, that has been recently revealed at the molecular level through transcriptome profiling and functional characterization of macrophages from different organs [63], [64].

Although the mouse is a rather resistant model to analyze systemic inflammation after Ad injection compared to rats [65], non-human primates [3] and humans [5], [6], some clinical aspects of virus-induced systemic toxicity are well reproduced in mice. One of the clinically relevant signs of systemic Ad-induced toxicity observed is thrombocytopenia. Intravascular administration of Ads was shown to trigger severe thrombocytopenia in all animal models analyzed [3], [4], [66], [67]. Remarkably, earlier studies showed that thrombocytopenia does not ensue after administration of Ad into spleenectomized mice [66], suggesting that virus interaction with cells in the spleen may be mechanistically linked to triggering this deleterious host response. Prolonged retention of PMNs in tissues leads to collateral tissue damage due to local neutrophil degranulation and release of cell-damaging proteases, thus exacerbating inflammation and contributing to the organ failure. Our findings might prove helpful for researchers developing therapeutic protocols for translational use of gene transfer vectors based on Ad. Our finding that a combination of the pharmacological inhibition of complement activation and IL-1RI-signaling deficiency reduces PMN recruitment to the site of infection and spares the virus-containing cell population provides the rationale for reducing damage to host cells after intravascular administration of adenovirus vectors by using pharmacological intervention. Specifically, the combination of complement inhibitors [50] with clinically-approved inhibitors of IL-1RI signaling, e.g. recombinant IL-1RA “Anakinra” [68], [69], may prove useful for both modulating systemic toxicity of Ad after its intravascular administration as well as under other conditions where initial pro-inflammatory stimuli are produced by tissue residential macrophages that recruit activated PMNs and cause collateral tissue damage.

## Materials and Methods

### Ethics statement

This study was carried out in strict accordance with the recommendations in the Guide for the Care and Use of Laboratory Animals of the National Institutes of Health. The protocol was approved by the Institutional Animal Care and Use Committee of the University of Washington, Seattle, WA, protocol number 4148-01, and all efforts were made to minimize suffering.

### Experimental animals

C57BL/6 mice were purchased from Charles River, Wilmington, MA. *Cxcr1* ^−/−^, *Cxcr2* ^−/−^, *C3* ^−/−^, *Il6* ^−/−^, and *Il1r1* ^−/−^ mice were purchased from Jackson Laboratory. *Il1a^−/−^*, *Il1a^−/−^b^−/−^* mice were described in [70]. *Il1b^−/−^* mice were described in [71]. All mice were on C57BL/6 genetic background, matched by age and housed in specific-pathogen-free facilities.

### Viruses

The replication-defective Ad5-based vectors, HAdv5 (Ad5L), Ad5/35S, and Ad-ts1 HAd2 mutant were previously constructed and described in detail elsewhere [8], [32], [72], [73]. For Ad amplification, 293 cells were infected under conditions that prevented cross-contamination. Viruses were banded in CsCl gradients, dialyzed and stored in aliquots as described earlier [73]. Ad genome titers were determined by OD_260_ measurement. For each Ad used in this study, at least two independently prepared virus stocks were obtained. Each produced virus stock was tasted for endotoxin contamination using *Limulus* amebocyte lysate Pyrotell (Cape Cod Inc, Falmouth, MA). For *in vivo* experiments, only virus preparations confirmed to be free of endotoxin contamination were used.

### Adenovirus delivery and estimation of the effective virus dose per MZ macrophage *in vivo*

Unless otherwise specified, mice were injected with 10^10^ virus particles in 200 µl of phosphate buffered saline (PBS) via tail vein infusion. At indicated times, mice were sacrificed and organs were harvested for further analyses. Previously published data shows that the frequency of marginal zone macrophages in the mouse spleen is 5.1% of splenocytes [74]. According to our earlier data on the absolute amount of Ad particles trapped in the spleen [10], we estimated that if mice are injected with a dose of 10^10^ virus particles, each marginal zone macrophage will receive between 50 and 100 virus particles per cell. We experimentally determined that an average mouse spleen possesses 2.5×10^8^ nucleated cells and yields on average 1 mg of total genomic DNA. Considering that the cumulative number of MZMφ is around 5% [74], the total average number of MZMφ cells in a mouse spleen is approximately 1.25×10^7^. Our quantitative real-time PCR analysis shows that upon the injection of mice with Ad at a dose of 10^10^ virus particles per mouse, the total number of Ad genomes accumulated in the liver 30 min after virus injection is 7×10^5^ per µg of splenic DNA, or 7×10^8^ per whole spleen. By dividing 7×10^8^ (the number of Ad genomes per whole spleen) by 1.25×10^7^ (the average number of MZMφ) we find a maximum actual dose of around 50 Ad particles per each MZMφ cell.

### Proteome Profiler Antibody arrays

A “Proteome Profiler antibody array: Mouse Cytokine Array Panel A” (#ARY006, R&D System) was used, according to the manufacturer's instructions. Each spleen was homogenized in 2 ml of sample solution, and 1 ml (1/2 spleen) was used to incubate with each membrane on a rocking platform overnight [31]. Membranes were developed with ImmunoStar HRP-sustrate (BioRad, #1705041).

### Antibodies and other materials

Propidium iodide was purchased from Sigma-Aldrich, (St.Louis, MO USA), Cat. #81845, antibodies from Abcam: biotinylated anti-Ad-Hexon (#ab34374, final dilution 1/100), anti-Ad5 (#ab6982, final dilution 1/50); Antibodies from BMA: anti-MARCO (BMA, #T2026, 2 ug/ml), Antibodies from BD: anti-IgM (#553405, 1 ug/ml), FITC-labeled anti-GR-1 (#553127), FITC-labeled anti-Ly-6G (#551460); R-PE-labeled anti-7/4 (Serotec MCA771PE Clone 7/4). Antibodies against Secondary antibodies and reagents were from Jackson Immunoresearch: Cy2 or Cy3-labeled streptavidin, or donkey anti-rat or rabbit antibodies, Cy2-, Cy3- or HRP-labeled. AMD3100 compound was purchased from Sigma-Aldrich and suspended in phosphate–buffered saline (PBS)/bovine serum albumin (BSA), and injected intraperitoneally at 50 mg per kg of mouse body weight. Complement inhibitor proteins CR2-Crry [49] and CR2-fH [50] were prepared as previously described.

### Flow cytometry cell analysis

Splenocytes, bone marrow mononuclear cells, and peripheral blood were harvested from mice at indicated time points after adenovirus administration and stained by incubation with FITC-labeled anti-Ly-6G and PE-labeled anti-7/4 primary monoclonal antibodies for 30 minutes at 4°C in triplicate. Next, cells were washed and analyzed by fluorescence-activated cell sorting on FACSCalibur machine (Becton Dickinson, San Jose, CA). Prior to flow cytometry analysis, red blood cells were lysed in all samples and nucleated cells were pelleted, washed, and processed as described above.

### Immunofluorescent and immunohistochemical analyses of tissues

Mice were anaesthetized, and spleens and livers were collected, frozen in O.C.T. compound and stored at −80°C until processed. Five consecutive 6–8 µm sections at 4 depth levels in the spleen and liver were cut, air dried, fixed for 10 minutes in acetone at −20°C, air dried for at least 4 hours, re-hydrated in TBS for one hour, blocked in 2% N.S. for 1 hour and incubated with primary antibodies overnight at 4°C with or without 0.1% saponin depending on the antigen. Then, sections were incubated with HRP-labeled secondary antibodies for 1 hour. Slides were developed with ImmPact DAB or NovaRed substrates (Vector Laboratories), air dried, mounted, and analyzed on a Leica microscope. For immunofluorescence staining, slides were immediately mounted after washing with the secondary antibodies. Images were taken using a CCD camera-equipped Leica dual light fluorescent microscope. Four representative images were taken for each section cut from tissues at at least 3 depth levels for quantitative processing using MetaMorph 7.8.1.0 software under interactive threshold settings to define positive staining on the image and applied universally to all images in analyzed experimental groups.

### Statistical analyses

Statistical analysis in each independent experiment was performed with using one-way ANOVA followed by Newman-Kleus *post-hoc* test with GraphPad Prism 5.02 software. Data are reported as mean ± standard deviation. *P*<0.05 was considered statistically significant.

### Accession numbers for genes and proteins used in the study

All accession numbers are provided as shown in Swiss-Prot database: mouse interleukin 1 alpha (IL-1β) accession number P01582; mouse interleukin 1 beta (IL-1β) accession number P10749; mouse interleukin 1 receptor type I (IL-1RI) accession number P13504; mouse complement component C3 (C3) accession number P01027; mouse growth regulated alpha protein (CXCL1) accession number P12850; mouse C-X-C chemokine 2 (CXCL2) accession number P10889; mouse C-X-C chemokine receptor type 1 (CXCR1) accession number Q810W6; mouse C-X-C chemokine receptor type 2 (CXCR2) accession number P35343; mouse interleukin 6 (IL-6) accession number P08505.

## Supporting Information

Figure S1 **MARCO^+^ marginal zone cells are eliminated from the spleen after sequestering Ad from the blood in a dose-dependent manner.** Immunohistochemical analysis of MARCO^+^ marginal zone macrophages on sections of spleen at 24 after Ad administration at indicated doses (virus particles per mouse). Spleens of mice injected with the virus were harvested and stained with MARCO-specific antibodies. Sections were counter-stained with hematoxylin to visualize splenic anatomical compartments. Mock – spleen sections were prepared from mice injected with saline only. Representative fields are shown. N = 5.
(TIF)
