# Supplementary figures and images for: IL-1α and Complement Cooperate in Triggering Local Neutrophilic Inflammation in Response to Adenovirus and Eliminating Virus-Containing Cells
Source: PLoS Pathog. 2014 Mar 20;10(3):e1004035. doi: 10.1371/journal.ppat.1004035 (PMC3961377; doi:10.1371/journal.ppat.1004035)

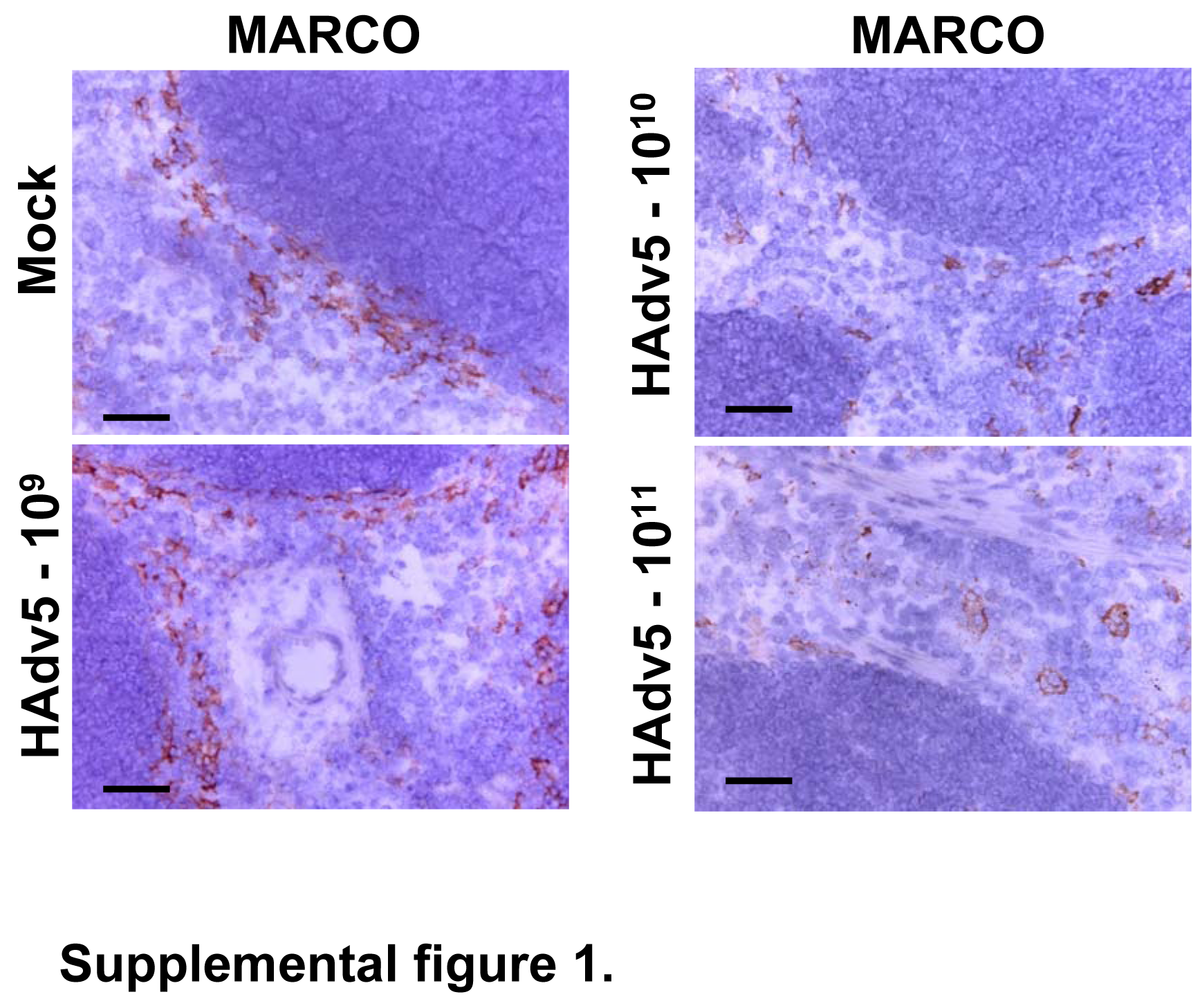

Supplement: Figure S1 — MARCO+ marginal zone cells are eliminated from the spleen after sequestering Ad from the blood in a dose-dependent manner. Immunohistochemical analysis of MARCO+ marginal zone macrophages on sections of spleen at 24 after Ad administration at indicated doses (virus particles per mouse). Spleens of mice injected with the virus were harvested and stained with MARCO-specific antibodies. Sections were counter-stained with hematoxylin to visualize splenic anatomical compartments. Mock – spleen sections were prepared from mice injected with saline only. Representative fields are shown. N = 5. (TIF) [file ppat.1004035.s001.tif]
